# A polymorphism in a phosphotyrosine signalling motif of CD229 (Ly9, SLAMF3) alters SH2 domain binding and T‐cell activation

**DOI:** 10.1111/imm.12513

**Published:** 2015-09-13

**Authors:** Stefanie Margraf, Lee I. Garner, Timothy J. Wilson, Marion H. Brown

**Affiliations:** ^1^ Sir William Dunn School of PathologyUniversity of OxfordOxfordUK; ^2^ Present address: Cambridge Institute for Medical ResearchUniversity of CambridgeCambridgeUK

**Keywords:** CD229, signalling, SLAM, systemic lupus erythematosus, T cell

## Abstract

Signalling lymphocyte activation molecule (SLAM) family members regulate activation and inhibition in the innate and adaptive immune systems. Genome‐wide association studies identified their genetic locus (1q23) as highly polymorphic and associated with susceptibility to systemic lupus erythematosus (SLE). Here we show that the Val_602_ variant of the non‐synonymous single nucleotide polymorphism (SNP) rs509749 in the SLAM family member CD229 (Ly9, SLAMF3) has a two‐fold lower affinity compared with the SLE‐associated Met_602_ variant for the small adaptor protein SAP. Comparison of the two variants in T‐cell lines revealed the Val_602_ variant to be significantly more highly expressed than CD229 Met_602_. Activation was diminished in cells expressing CD229 Val_602_ compared with CD229 Met_602_ as measured by up‐regulation of CD69. There was no correlation between homozygosity at rs509749 and activation in peripheral blood mononuclear cells from healthy donors. These findings identify potential mechanisms by which a single SNP can perturb fine‐tuning in the immune system with significant functional consequences.

**Abbreviations**
FCS: fetal calf serum
HA: haemagglutinin
ITSM: immunoreceptor tyrosine‐based switch motif
JC20: Jurkat clone 20
MCS: multiple cloning site
PBMC: peripheral blood mononuclear cell
SAP: signalling lymphocyte activation molecule–associated protein
SH2 domain: Src homology 2 domain
shRNA: small hairpin RNA
SLAM: signalling lymphocyte activation molecule
SLE: systemic lupus erythematosus
SNP: single nucleotide polymorphism

## Introduction

Signalling lymphocyte activation molecule (SLAM) family receptors are expressed on haematopoietic cells and include CD150 (SLAM, SLAMF1), CD48 (SLAMF2), CD229 (Ly9, SLAMF3), CD244 (2B4, SLAMF4), CD84 (SLAMF5), CD352 (NTB‐A, SLAMF6), CD319 (CRACC, CS1, SLAMF7), BLAME (SLAMF8) and SLAMF9 (CD84H1, CD2F10).^1^, ^2^ With the exception of CD229, SLAM family receptors contain two extracellular immunoglobulin superfamily domains. CD229 has four immunoglobulin superfamily domains because of a tandem repeat of the distal V and C2 domains. CD229 interacts homotypically^3^ via its N‐terminal V domain as is typical for SLAM family receptors.

SLAM family receptors signal through one or more intracellular immunoreceptor tyrosine‐based switch motifs (ITSMs).^4^, ^5^ This motif (TxYxxI/V) binds SH2 domains of the adaptor molecules, signalling lymphocyte activation molecule–associated protein (SAP; SH2D1A) and EAT‐2 (SH2D1B) in a tyrosine‐phosphorylation‐dependent manner.^4^, ^6^, ^7^, ^8^ The importance of SAP became clear from studying patients with X‐linked lymphoproliferative disease who exhibit an abnormal immune response to Epstein–Barr virus infection. This rare immunodeficiency is typically associated with the absence or mutation of SAP.^1^, ^2^, ^7^, ^9^ SAP is expressed in leucocytes and links the kinase Fyn with SLAM family receptors in an atypical SH2–SH3 domain interaction.^10^, ^11^, ^12^ Absence of functional SAP in T cells and natural killer cells severely compromises cellular immunity.^8^, ^9^, ^13^, ^14^ SLAM family receptors manifest activating and inhibitory effects, and the term ‘switch motif’ is derived from the dual specificity of ITSMs for activating and inhibitory SH2‐domain‐containing proteins. The switch from activation to inhibition is observed in the absence of SAP.^4^ Direct competition between SAP and SH2‐domain‐containing phosphatases for ITSMs has been proposed as a molecular mechanism for inhibition.^4^, ^15^

The cytoplasmic tail of CD229 contains two ITSMs and an interaction between CD229 and SAP has been shown.^16^ In common with all the SLAM family receptors that signal through ITSMs, activating effects of CD229 are dependent on SAP in primary human cells.^17^ Studies with genetically manipulated mice lacking SLAM family adaptors revealed evidence of both activating and inhibitory effects of CD229.^18^, ^19^, ^20^, ^21^

CD229 and other SLAM family members have been linked with susceptibility to systemic lupus erythematosus (SLE), a complex autoimmune disease.^22^, ^23^ The human SLAM locus of 1q23 is polymorphic and is syntenic with the SLE‐associated *Sle1* locus in mice.^24^ A linkage study of families based on the SLAM locus identified an association between susceptibility to SLE and a single nucleotide polymorphism (SNP) rs509749 that causes a non‐synonymous exchange in exon 8 of CD229.^23^ Susceptibility correlated with a Val_602_ (TVYAQV) to Met_602_ (TMYAQV) switch in the first ITSM of CD229.^23^ We have previously characterized differences in fine specificity of ITSMs, which correlated with functional data.^6^ Here we compare the two variants of Ly9 and reveal differences in binding properties and functional effects that can explain how the association came to be identified.

## Material and methods

#### Cells and antibodies

Media were purchased from Sigma‐Aldrich (St Louis, MO). Jurkat Clone 20 (JC20) and HEK‐293T cells were grown in RPMI‐1640 and Dulbecco's modified Eagle's medium (4·5 g/l glycerol, 110 mm sodium pyruvate), respectively, supplemented with 2 mm glutamine, 10% fetal calf serum (FCS), 100 U/ml penicillin and 100 μg/ml streptomycin. Puromycin (1 μg/ml) was added to maintain protein and small hairpin (sh)RNA expression in stable cell lines. Peripheral blood mononuclear cells (PBMCs) were isolated from the blood of healthy donors by Ficoll density gradient centrifugation and frozen at −80° in FCS containing 10% DMSO.

Antibodies used in this study were: anti‐T‐cell receptor *αβ* clone IP26 (phycoerythrin‐conjugated; Biolegend, San Diego, CA), anti‐CD3*ε* clone Leu‐4 (phycoerythrin‐conjugated; Becton Dickinson, Franklin Lakes, NJ), anti‐CD229 clone HLy9.1.25 (FITC and un‐conjugated; AbD Serotec, Kidlongton, UK), anti‐CD69 (allophycocyanin‐conjugated; Invitrogen, Calrsbad, CA), anti‐CD3*ε* clone UCHT1 (AbD Serotec and eBioscience, San Diego, CA) and anti‐CD28 clone CD28.2 (Biolegend).

#### Genotyping

Genomic DNA was purified from 4 × 10^6^ PBMCs using the PureLink^®^ Genomic DNA Kit (Life Technologies, Grand Island, NY). DNA of exon 8 of CD229 encoding rs509749 was amplified by PCR using the primers 5′‐ACTCTGTGCTCTCCCAAGGA‐3′ and 5′‐TAGTGGCCCAAGAATGAGAGCTG‐3′ sequenced using primer 5′‐TGCACAAGCCCATCAGTGG‐3′ and analysed with geneious 6·1·5 (Biomatters Limited, Auckland, New Zealand).

#### Plasmid constructs

The bicistronic pU6‐MCS‐EF1‐GOI‐IRES‐Puro^R^ lentiviral vector was created by modifying the plasmid pU6‐shRNA‐EF1‐GOI‐IRES‐Puro^R^.^25^ The U6 promoter sequence amplified using the primers 5′‐GAGCAATTGGGCAGGAAGAGGGCCTAT‐3′ and 5′‐GAGAATTCGTTTAAACTTAATTAAGGCGCGCCGTCCTTTCCACAAGATATATAAAGC‐3′ was inserted into *Eco*RI‐cut pU6‐shRNA‐EF1‐GOI‐IRES‐Puro^R^ creating a multiple cloning site (MCS) downstream of the U6 promoter containing the restriction sites 5′□3′*Asc*I, *Pac*I, *Pme*I and *Eco*RI. The shRNAs generated by slow annealing of the 5′‐TAAGCGCGCTTTGTAGGATTCGTTTCAAGAGAACGAATCCTACAAAGCGCGCTTTTTTG‐3′ sequence with 5′‐AATTCAAAAAAGCGCGCTTTGTAGGATTCGTTCTCTTGAAACGAATCCTACAAAGCGCGCTTAAT‐3′ and of the 5′‐CGCGCCGTAGCGTTTCCTCCTCGAAATTTTCAAGAGAAATTTCGAGGAGGAAACGCTATTTTTTG‐3′ sequence with 5′‐AATTCAAAAAATAGCGTTTCCTCCTCGAAATTTCTCTTGAAAATTTCGAGGAGGAAACGCTACGG‐3′ creating the scrambled shRNA (shCTR) and the 3′‐untranslated region targeting CD229 shRNA [shCD229 – hairpin sequence (underlined) as from Sigma‐Aldrich, mission shRNA], respectively, were cloned 3′ of the U6 promoter using the *Pac*I and *Eco*RI (shCTR) or *Asc*I and *Eco*RI (shCD229) sites. The CD229 sequence (NM_002348) with the *Eco*RI restriction sites (GAATTC) destroyed by site directed mutagenesis (GAATTT) was inserted into pU6‐MCS‐EF1‐GOI‐IRES‐Puro^R^ via *Spe*I and *Sma*I. HA constructs were generated by cloning the secretion signal peptide from pHLsec followed by a haemagglutinin (HA)‐tag 26 into the pU6‐MCS‐EF1‐GOI‐IRES‐Puro^R^ using forward 5′‐TAGACTAGTATGGGCATCCTTCCCAGC‐3′ and reverse 5′‐TAGCCCGGGTTGATCAGGTTTAAACATAGCGTAGTCTGGGACGTCG‐3′ oligos creating a *Pme*I–*Xma*I insertion site. The DNA fragment of CD229 encoding the short extracellular linker, the transmembrane and cytoplasmic domain (residues Ser^434^ to Thr^655^) was inserted in‐frame via *Pme*I and *Xma*I to generate pU6‐MCS‐EF1‐HACD229^434‐656^‐IRES‐Puro^R^.

#### Lentiviral transduction

Lentiviral particles were produced by transfecting HEK‐293T cells at 60% confluence with a 4 : 2 : 1 ratio of the lentiviral expression: psPAX2 packaging: pMD2.G envelope vectors at a 2·5 : 1 ratio of FuGENE^®^ (Promega, Madison, WI): DNA in Dulbecco's modified Eagle's medium and 1% FCS for 16 hr at 37°. The medium was replaced with RPMI‐1640 and 10% FCS and supernatant containing viral particles was collected after 48 hr and filtered (0·45 μm). JC20 cells (0·7 × 10^6)^ were resuspended in 2 ml virus‐containing supernatant, supplemented with 5 μg/ml polybrene and centrifuged in a 12‐well flat bottom plate at 30° at 1350 ***g*** for 1·5 hr. Supernatant was replaced after 16 hr by RPMI‐1640, 10% FCS and puromycin (1 μg/ml).

#### T‐cell activation and flow cytometry

Jurkat T cells (5 × 10^4^) or PBMCs (0·2 × 10^5^) were plated in each well of 96‐well flat‐bottomed plates pre‐coated with the indicated concentration of anti‐CD3*ε* and stimulated for 6 hr or 16 hr, respectively. Surface molecules were stained with the appropriate antibodies directly before and after stimulation and expression levels were analysed using the BD FACSCalibur™ and Beckman Coulter CyAn™ ADP Analyzer. Cells were analysed based on live, single cells according to forward light scatter/side scatter characteristics unless stated otherwise. Data were evaluated using flowjo, MS excel and graphpad prism 6 software.

#### Western blot analysis

Western blotting was performed as previously described.^6^ Briefly 10^7^ Jurkat T cells were lysed in 200 μl Triton X‐100 buffer and 2·5 × 10^5^ to 7·5 × 10^5^ cells were resolved by SDS–PAGE under reducing conditions. Protein bands were detected by the LI‐COR Odyssey Sa system after developing with rabbit anti‐SAP antibody (clone FL‐128 Santa Cruz Biotechnology (Santa Cruz, CA)), mouse anti‐phosphotyrosine (clone PT‐66, Sigma, St Louis, MO), goat anti‐HA (biotinylated, Vector Laboratories) or anti‐*β*‐actin (clone AC‐15, Santa Cruz Biotechnology) followed by the appropriate secondary antibody or fluorophore‐conjugated streptavidin (IRDye 680LT‐conjugated anti‐rabbit IgG, IRDye 800 CW anti‐mouse IgG, IRDye 680LT‐conjugated anti‐mouse IgG, IRDye 800 CW Streptavidin; all LI‐COR Biosciences, Lincoln, NE). Quantification of protein expression was performed using LI‐COR odyssey sa software version 1.0.

#### Surface plasmon resonance

Synthetic peptides all biotinylated at the N‐terminus for human CD229M (GENTMpYAQVFN), CD229V (GENTVpYAQVFN), CD244‐ITSM1 (EFLTIpYEDVKD), CD244‐ITSM2 (GGSTIpYSMIQS) and Fc*γ*RIIb‐ITIM (ADKVGAENTITpYSLLMHPDA) were from Peptide Protein Research Ltd, Fareham, Hampshire, UK.^27^ Recombinant human proteins, full length SAP,^27^ and SHIP SH2 domain from SHIP‐1 [SHIP(SH2)]^28^ were produced in‐house and purified on the day of the experiment.^6^ Protein concentrations were determined by absorbance at 280 nm using theoretical extinction coefficients.^27^, ^28^ Peptide–protein affinities were measured using the BIAcore 3000 (GE Healthcare, Chalfont St Giles, UK). A range of concentrations of purified, recombinant proteins was passed over peptides immobilized on a streptavidin‐coated CM5 chip [~30 response units (RUs)] in 10 mm HEPES, pH 7·4, 150 mm KCl, 10 mm EDTA, 0·005% volume/volume Tween 20. The signal in a reference cell coated with streptavidin was subtracted. Equilibrium binding dissociation constants were calculated using biaevaluation and graphpad prism 6 software by fitting the equilibrium data using a one‐site binding curve.^6^

#### Statistical analysis

*P*‐values were calculated as indicated in the figure legends using graphpad prism 6 software. For comparison of HA expression levels, HA‐CD229 Met_602_ (HAM) and HA‐CD229 Val_602_ (HAV) data sets after stimulation with 0·5 μg/ml anti‐CD3*ε* from five experiments were merged and classified according to their expression levels. Cells below 25% of total HA‐expression were defined as HA^lo^, above 75% as HA^high^ and intermediate cells as HA^int^. Medians of CD69‐staining intensities were used for performing paired *t*‐test analysis.

## Results

### The SLE‐associated variant of CD229 ITSM1 (Met_602_) binds more strongly than the Val_602_ variant to SAP

We first investigated the effect of rs509749 on interactions of the CD229 ITSM. As it was reported that rs509749 affected T‐cell populations^23^ we focused on measuring the interaction between CD229 and the adaptor SAP because both are expressed in T cells^5^ and an interaction of CD229 ITSMs with SAP has been shown.^16^ We measured the interaction between SAP and the ITSMs of the two variants of CD229 by surface plasmon resonance at 37°. Increasing concentrations of soluble monomeric recombinant human SAP were injected over immobilized peptides representing the CD229 Val_602_ or Met_602_ ITSMs phosphorylated on the tyrosine residues (Fig. 1a). Plotting the equilibrium binding data, we observed a two‐fold higher K_D_ for binding of SAP for the variant CD229 Val_602_ (K_D_ = 2·8 μm) compared with the risk allele, Met_602_ of CD229 (K_D_ = 1·5 μm) (Fig. 1b). The same two‐fold weaker affinity for the allele Val_602_ was consistently observed in three separate experiments (Table 1). ITSM peptides of the same length from CD244 served as a control for protein activity (Table 1).^6^

**Figure 1:**
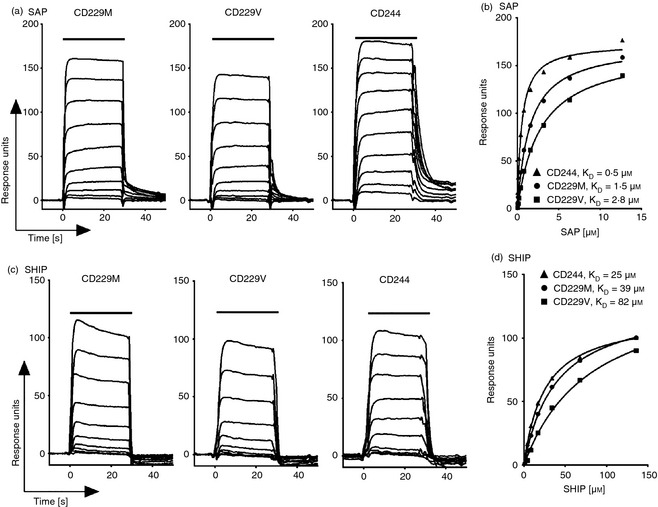
The systemic lupus erythematosus (SLE) associated immunoreceptor tyrosine‐based switch motif (ITSM1; Met_602_) of CD229 binds more strongly than the variant (Val_602_) to signalling lymphocyte activation molecule‐associated protein (SAP). Surface plasmon resonance sensorgrams (a, c) and equilibrium binding curves (b, d) of two‐fold increasing concentrations of SAP or SHIP(SH2) (top concentrations of SAP and SHIP were 12·7 μm and 136 μm, respectively) injected for 30 s at 37° over immobilized phosphorylated peptides representing the two CD229 ITSMs (T**M**pYAQV = CD229M or (T**V**pYAQV = CD229V) and CD244 ITSM1.

**Table 1:** Equilibrium dissociation constants for SHIP and SAP binding to CD229 and CD244 immunoreceptor tyrosine‐based switch motif (ITSM)

	SAP			SHIP(SH2)		
	*K*_D_	Range	*n*	*K*_D_	Range	*n*
CD229M	1·7	1·5–2·0	3	>50	23–92	3
CD229V	3·4	2·8–4·4	3	100	82–110	3
CD244‐ITSM1	0·5		1	25		1
CD244‐ITSM2	0·09		1	2·4		1
Fc*γ*RIIb‐ITIM	N.A.		1	0·36		1

Mean *K*_D_ values (μm) for soluble SAP and SHIP(SH2) binding to immobilized phosphorylated CD229‐ITSM1, CD244‐ITSM1, CD244‐ITSM2 and Fc*γ*RIIb‐ITIM peptides as analysed by surface plasmon resonance at 37°; range and number (n) of independent experiments.

We compared binding of SAP to tyrosine phosphorylated peptides of the same length representing the CD229 ITSM containing the rs509749 SNP (TV/MpYAQ) and the more distal ITSM (TIpYCS). SAP bound to the second non‐polymorphic sequence (TIpYCS) with up to an order of magnitude greater strength (K_D_ = 0·1 μm at 37°C; *n* = 3 data not shown).

### The CD229 Met_602_ to CD229 Val_602_ substitution does not alter specificity for an SH2 domain from an inhibitory phosphatase

The ITSMs have dual specificity for the adaptor proteins and for SH2‐domain‐containing inhibitory enzymes including the inositol phosphatase, SHIP.^6^, ^29^ To test whether the Val_602_ to Met_602_ modification in ITSM1 in CD229 alters specificity, we measured binding of the SH2 domain from the phosphatase SHIP‐1 to both variants. Binding of the SHIP SH2 domain was detected but the affinity was at least an order of magnitude weaker than SAP binding (Fig. 1). The Val_602_ to Met_602_ variation did not alter specificity for an activating adaptor SH2 domain compared with an SH2 domain from an inhibitory enzyme. Equilibrium dissociation constants for binding of SHIP SH2 domain to CD244 ITSM1 and Fc*γ*RIIb ITIM are within the range for published data^6^ and show that the protein is active (Table 1).

### CD229 Val_602_ is expressed more highly than the SLE‐associated CD229 Met_602_ variant

To examine how rs509749 SNP affects the surface expression of CD229 receptor in cells, we expressed the Val_602_ and Met_602_ variants in Jurkat cells. Jurkat cells express CD229 and SAP (Fig. 2a and see Fig. 4b). We used a bicistronic vector to down‐modulate endogenous CD229 by targeting its 3′ UTR and simultaneously express CD229 Val_602_ or CD229 Met_602_ under the control of the pEF1*α* promoter. Flow cytometry confirmed that endogenous CD229 was reduced by transduction of shCD229 and not by a control scrambled shRNA construct (Fig. 2a,b). Transduced CD229 Val_602_ and CD229 Met_602_ were concomitantly expressed (Fig. 2a,b). The allele, CD229 Val_602_ was consistently expressed at a higher level compared with the CD229 Met_602_ variant and both were more abundant at the cell surface than the original endogenous CD229 (Fig. 2a,b).

**Figure 2:**
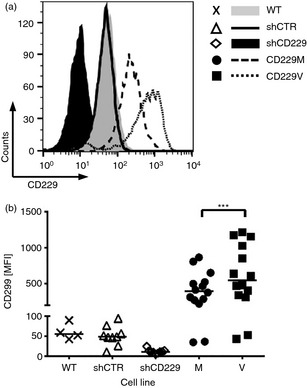
CD229 Val_602_ is expressed more highly than the systemic lupus erythematosus (SLE) ‐associated CD229 Met_602_ variant. (a, b) Endogenous CD229 expression of wild‐type Jurkat JC20 cells (WT) and JC20 cells stably transduced with control small hairpin (sh) RNA (shCTR) or CD229 3′ untranslated region (UTR) shRNA (shCD229). CD229 expression on JC20 cells stably transduced with a bicistronic vector to re‐express CD229 Met_602_ (CD229M, M) or CD229 Val_602_ (CD229V, V). (b) Data from two separately generated cell lines analysed on seven different days (M, V) and from one cell line each, measured on nine (shCTR, shCD229) or four (WT) different days. Horizontal lines denote the median value. ***P < 0·001, Wilcoxon signed‐rank test.

### Activation through CD3 is diminished in Jurkat T cells expressing CD229 Val_602_ compared with CD229 Met_602_

The biochemical and expression data revealed two differences between the CD229 variants; weaker binding to SAP and higher expression at the cell surface by the CD229 Val_602_. We compared the functional effects of endogenous CD229 and the two variants in the Jurkat cells by measuring up‐regulation at the cell surface of the activation marker CD69 after anti‐CD3*ε* stimulation. There was no significant difference in the dose‐dependent response to anti‐CD3*ε* cross‐linking between cell lines expressing endogenous CD229 (shCTR), shCD229, CD229 Met_602_ or CD229 Val_602_ (Fig. 3a). In these experiments there was no significant difference between the levels of CD3 or CD229 in CD229 Met_602_ or CD229 Val_602_ expressing cell lines (Fig. 3b). These data show that the manipulation of CD229 expression has no deleterious effect on the cells.

**Figure 3:**
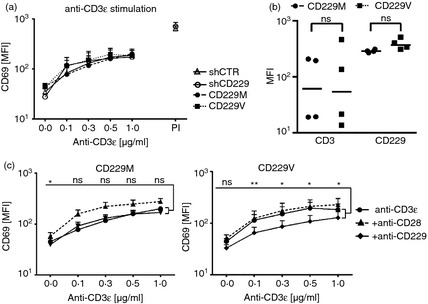
Activation through CD3 is diminished in Jurkat T cells expressing CD229 Val_602_ compared with CD229 Met_602_. (a) CD69 expression on stably transduced JC20 cells stimulated for 6 hr with plate‐bound anti‐CD3*ε*. PMA/Ionomycin (P/I) serves as a positive control. (b) CD3 and CD229 expression levels. Lines indicate median and statistics show *P*‐value of two‐tailed *t*‐test. (c) CD69 expression levels of stable transduced JC20 cells stimulated for 6 hr with plate‐bound anti‐CD3*ε* alone or in combination with soluble anti‐CD28 or anti‐CD229. Paired *t*‐test was used to calculate differences between anti‐CD3*ε* and anti‐CD229 stimulation. ns: non‐significant, *P < 0·05, **P < 0·01. Results represent mean of two experiments. In each experiment two independently generated CD229M and CD229V cell lines were analysed. Error bars in (a) and (c) represent +SEM.

To investigate specific signalling effects of the two CD229 variants, we used a monoclonal antibody that has been shown to be effective in cross‐linking and triggering through CD229.^30^ We compared the effects of cross‐linking CD3 and CD229 or CD3 and CD28. Co‐stimulation by CD28 was observed in CD229 Met_602_ but not CD229 Val_602_ cells (Fig. 3c). There was also a difference in response between CD229 Met_602_ and CD229 Val_602_ cells when using anti‐CD229. Cross‐linking CD229 Val_602_ had an inhibitory effect on CD3‐induced CD69 expression levels, which were unaltered in CD229 Met_602_ cells. These data revealed a functional difference between CD229 Val_602_‐ and CD229 Met_602_‐expressing cells.

### The cytoplasmic tail of CD229 Val_602_ is more inhibitory than the SLE‐associated CD229 Met_602_ cytoplasmic tail

To focus on detecting a potential signalling difference between CD229 Val_602_ and CD229 Met_602_, the extracellular region of the receptor was replaced by an HA‐tag. Flow cytometry confirmed down‐modulation of the endogenous full‐length receptor by its shRNA and concomitant expression of HA‐tagged CD229 (Fig. 4a). Similarly to the full‐length receptors, the HA‐CD229 Val_602_ (HAV) was more highly expressed than HA‐CD229 Met_602_ (HAM) (Fig. 4a,b) confirming that this expression effect was due to the cytoplasmic region. There were equal levels of SAP in both cell lines and receptors were phosphorylated to the same extent as judged by Western blot analysis (Fig. 4b).

**Figure 4:**
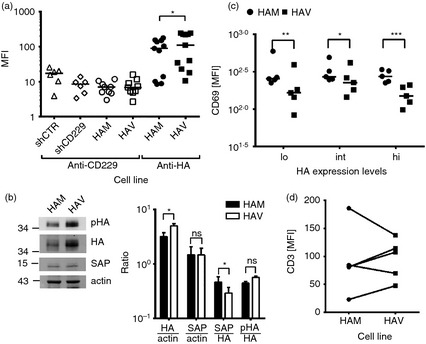
The cytoplasmic tail of CD229 Val_602_ is more inhibitory than the systemic lupus erythematosus (SLE) associated CD229 Met_602_ cytoplasmic tail. (a) CD229 or haemagglutinin (HA) expression levels on stably transduced Jurkat JC20 cells expressing control small hairpin (sh) RNA (shCTR), CD229 targeting shRNA (shCD229) or a CD229 variant with the extracellular domain replaced by an HA‐tag (HAM or HAV). *P = 0·01 based on a Wilcoxon signed‐rank test. (b) Western blot (center panel) and ratio between proteins (right panel) of HA‐CD229 Met_602_ (HAM) or HA‐CD229 Val_602_ (HAV) expressing JC20 cell lysates. Phosphorylated HA‐CD229 (pHA) was detected with mouse anti‐phosphotyrosine. Bars represent means of ratios +SEM of three independent experiments. *P < 0·05 (paired *t*‐test) (c) CD69 expression levels of HAM or HAV expressing Jurkat T cells after anti‐CD3*ε* stimulation with 500 μg/ml. Cells were grouped into low (lo) intermediate (int) and high (hi) HA‐CD229 expressing cells as described in the Materials and methods. Lines indicate median. *P < 0·05 **P < 0·01 ***P < 0·001 (paired *t*‐test of five independent experiments). (d) CD3 expression levels of HAM or HAV expressing Jurkat T cells before stimulation.

Cells expressing HA‐CD229 Val_602_ or HA‐CD229 Met_602_ were stimulated with anti‐CD3*ε* and analysed for up‐regulated CD69 expression by flow cytometry (Fig. 4c). We analysed low (< 25%), intermediate (25–75%) and high (>75%) HA‐CD229 expression levels for each experiment. Reduced activation by HA‐CD229 Val_602_‐ compared with HA‐CD229 Met_602_‐expressing cells was consistently observed (Fig. 4c). There was no correlation between reduced activation and TCR/CD3 or between TCR/CD3 and CD229 expression (Fig. 4d). These data suggest that altered signalling contributes to the reduced T‐cell response in HA‐CD229 Val_602_ cells.

### There is no correlation between expression of SLE‐associated CD229 Met_602_ and increased activation of healthy primary human cells

CD229 was up‐regulated on cells from SLE patients but the genotype of CD229 was not determined in that study.^17^ The A/G variants of rs509749 SNP are present in nearly equal measure (A = 55%, G = 45%) in the European population whereas in other populations, G is the major allele.^31^ We genotyped five healthy donors and identified two heterozygotes and three homozygotes, two CD229 Met_602_ and one CD229 Val_602_. We compared levels of expression of CD229 and CD3 on PBMCs from the five human donors (Fig. 5a,b). CD229 was expressed at low levels with no significant differences between the genotypes nor was there any evidence that CD229V was expressed more highly than CD229M on T blasts (Fig. 5a and data not shown). We stimulated PBMCs from the five donors with anti‐CD3*ε* and measured up‐regulation of CD69. The two heterozygous donors responded more strongly compared with the homozygotes. Increased responses by the heterozygotes did not correlate with CD3 levels, which were lower for the heterozygotes compared with the homozygotes.

**Figure 5:**
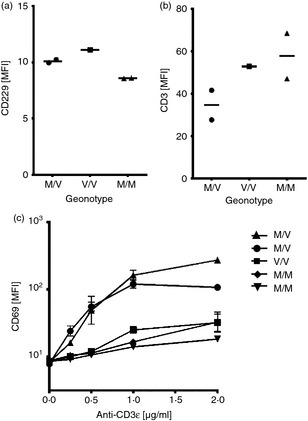
There is no correlation between expression of systemic lupus erythematosus (SLE) associated CD229 Met_602_ and increased activation of healthy primary human cells. (a) CD229 and (b) CD3 expression on primary peripheral blood mononuclear cells (PBMCs) from five healthy donors (values represent means of expression levels measured directly after PBMC isolation and before CD3 stimulation). (c) Percentage of CD69^+^ primary T lymphocytes after anti‐CD3*ε* stimulation of PBMCs. T cells were gated as T‐cell receptor *αβ*+. Data represent triplicates of one experiment.

## Discussion

The SNP rs509749 was associated with susceptibility of SLE and with altered T‐cell populations in a family‐based study.^23^ The molecular mechanism of genome‐wide associations with disease is rarely known. We identified biochemical and functional differences between two variants of CD229 that differ at rs509749, a non‐synonymous SNP that alters the sequence of the membrane proximal ITSM1 of CD229 from TMYAQV to TVYAQV.

The ITSMs in the variants of rs509749 in CD229 differ at the Tyr‐1 position. The amino acid at the Tyr‐1 position is not crucial but influences the three‐pronged binding mechanism of SH2 domains to ITSMs.^32^ The CD229 ITSM1 alleles differ only at the Tyr‐1 position and a two‐fold difference in affinity for SAP between CD229 Met_602_ (K_D_ = 1·7 μm) and CD229 Val_602_ (K_D_ = 3·4 μm) for SAP was measured. There was no evidence of a switch in specificity as suggested previously.^23^ A higher affinity of SAP for the membrane distal ITSM2 might explain why the polymorphism at Tyr‐1 of ITSM1 does not cause greater differences in CD229 signalling and impact on SLE.

The recombinant variant, CD229 Val_602_ was more highly expressed than the SLE‐associated CD229 Met_602_ in Jurkat T cells. Several SLE‐associated SNPs have been shown to change epigenetic imprints by modulating gene transcription.^33^ Transcriptional alterations can be excluded in the comparison of CD229 variants transduced into Jurkat cells because the promoter as well as coding regions are not native. Nevertheless messenger RNA and/or protein may be more stable for CD229 Val_602_ compared with CD229 Met_602_. Analysis of flow cytometric data indicated that CD229 Val_602_ was more readily internalized after CD3 monoclonal antibody‐induced activation compared with CD229 Met_602_, suggesting that slower internalization of CD229 Val_602_ is unlikely to explain the difference in expression (unpublished data). Significant differences in CD229 expression levels were not observed in PBMCs from healthy donors. There was up‐regulation of SLAM family receptors, CD229 and of CD352 (NTB‐A, SLAMF6) in T cells from patients with SLE compared with healthy controls.^17^ However, among the 11 SLE patients studied, there was a lower correlation between expression and disease severity for CD229 compared with CD352.^17^ The association between CD229 expression levels and SLE susceptibility is not straightforward.

An imbalance between stimulus strength, expression levels of receptor and signalling molecules can change the outcome of cellular activity, as has been shown for the SLAM receptor CD244 (2B4).^34^ Over‐expressing CD229 will alter the balance between receptor and intracellular adaptor. Increased expression correlated with an inhibitory phenotype suggesting that SAP, on which activation depends, may be limiting. Indeed, we found comparable expression levels of SAP in cell lines expressing the two CD229 variants, implying a lower adaptor to receptor ratio in cells expressing the CD229 Val_602_ variant. The lower affinity of SAP for the CD229 Val_602_ variant as well as the imbalance between adaptor and receptor might account for diminished activity of cells transduced with the SLE susceptible CD229 variant in comparison with CD229 Met_602_.

In conclusion, amplification of differences at the molecular and cellular levels between the CD229 variants might be sufficient to explain how rs509749 was identified as being associated with susceptibility to SLE specifically in a family‐based study.^23^, ^35^, ^36^

## Author contribution

SM designed and analysed functional experiments and wrote the manuscript. LIG and TJW performed and analysed the surface plasmon experiments. MHB directed the research and wrote the manuscript.

## Disclosure

There are no conflicts of interest.
